# Antibiotic Resistance Is Prevalent in an Isolated Cave Microbiome

**DOI:** 10.1371/journal.pone.0034953

**Published:** 2012-04-11

**Authors:** Kirandeep Bhullar, Nicholas Waglechner, Andrew Pawlowski, Kalinka Koteva, Eric D. Banks, Michael D. Johnston, Hazel A. Barton, Gerard D. Wright

**Affiliations:** 1 M.G. DeGroote Institute for Infectious Disease Research, Department of Biochemistry and Biomedical Sciences, McMaster University, Hamilton, Ontario, Canada; 2 Department of Biology, University of Akron, Akron, Ohio, United States of America; Cairo University, Egypt

## Abstract

Antibiotic resistance is a global challenge that impacts all pharmaceutically used antibiotics. The origin of the genes associated with this resistance is of significant importance to our understanding of the evolution and dissemination of antibiotic resistance in pathogens. A growing body of evidence implicates environmental organisms as reservoirs of these resistance genes; however, the role of anthropogenic use of antibiotics in the emergence of these genes is controversial. We report a screen of a sample of the culturable microbiome of Lechuguilla Cave, New Mexico, in a region of the cave that has been isolated for over 4 million years. We report that, like surface microbes, these bacteria were highly resistant to antibiotics; some strains were resistant to 14 different commercially available antibiotics. Resistance was detected to a wide range of structurally different antibiotics including daptomycin, an antibiotic of last resort in the treatment of drug resistant Gram-positive pathogens. Enzyme-mediated mechanisms of resistance were also discovered for natural and semi-synthetic macrolide antibiotics via glycosylation and through a kinase-mediated phosphorylation mechanism. Sequencing of the genome of one of the resistant bacteria identified a macrolide kinase encoding gene and characterization of its product revealed it to be related to a known family of kinases circulating in modern drug resistant pathogens. The implications of this study are significant to our understanding of the prevalence of resistance, even in microbiomes isolated from human use of antibiotics. This supports a growing understanding that antibiotic resistance is natural, ancient, and hard wired in the microbial pangenome.

## Introduction

The question of whether the extensive presence of resistance elements in microbes is primarily the result of human activity is controversial. Investigation of antibiotic resistance in bacteria from the Galapagos, a remote environment with limited human occupation and presumably low anthropogenic antibiotic exposure, revealed that acquired antibiotic resistance genes were absent in bacteria isolated from terrestrial animals [1]. Similarly, plasmids from bacterial collections that predate the antibiotic era are largely devoid of resistance elements [2], [3]. This suggests that the presence of antibiotics is an important selective force in evolution and spread of antibiotic resistance genes and can contribute significantly in altering the natural microbiota. In a survey of soil samples from the Netherlands spanning the pre-and post-antibiotic eras (1940–2008), an increase in the relative abundance of antibiotic resistance genes for major antibiotic families (ß-lactams, tetracyclines and macrolides) was observed in contemporary soil samples in comparison to pre-antibiotic era samples [4]. This body of evidence is consistent with the hypothesis that widespread resistance is a modern phenomenon linked to human use of antibiotics.

On the other hand, antibiotics and antibiotic biosynthetic pathways are believed to have evolved over millions of years suggesting that antibiotic resistance is an equally ancient phenomenon [5], [6], [7]. Indeed, we have recently shown that antibiotic resistance elements were abundant and diverse in ancient DNA dating from the Pleistocene (30,000 years ago) [8]. The concept of the antibiotic resistome predicts that resistance is the result of dynamic and competitive microbial interactions that pre-date human use of antibiotics [9], [10]. Consistent with this notion is our survey of contemporary soil actinomycetes that reported widespread multidrug resistance even in the absence of obvious human sources of antibiotics [11]. There have also been reports of antibiotic resistance in microorganisms isolated from extreme natural habitats including the deep terrestrial subsurface [12] and the deep ocean [13]; environments presumably largely absent of human influence. These studies support a hypothesis that resistance is an ancient and genetically rich natural phenomenon, deeply embedded in the microbial pangenome.

One of the challenges in measuring contemporary environmental resistance is rigorously ensuring the absence of anthropogenic sources of antibiotics as a selective pressure for the acquisition of ‘modern’ resistance genes. A survey of a diverse environmental bacterial population that has never been exposed to modern antibiotics and resistance genes would unequivocally establish the depth of the contemporary environmental resistome: Lechuguilla Cave offers a unique environment for such a study.

**Figure 1:**
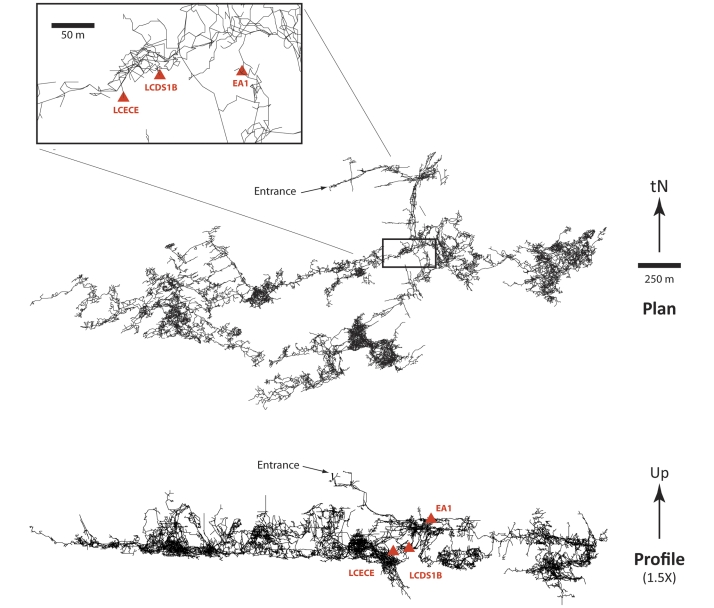
Plan and profile maps of Lechuguilla Cave, Carlsbad Caverns National Park, New Mexico. The sites where microbial strains were collected (LCECE, LCDS1 and LCEA1) are shown relative to the entrance and depth. tN represents true North on the plan, while the profile has an exaggerated vertical profile of 1.5×.

Lechuguilla Cave, located within Carlsbad Caverns National Park (USA), was formed in the Capitan Formation of the Delaware Basin by hypogenic (ascending water) sulfuric acid speleogenesis in the last 7 million years [14], [15], [16], resulting in the formation of a very large (>200 km) and deep (>500 m), maze-like cave system [17]. While the cave is still connected to the artesian aquifer that formed it (Figure 1), the majority of the cave became isolated as the aquifer dropped [18]. Due to its isolated hypogene nature, extensive cave development occurred primarily at −300 to −400 m, while the impermeable Yates Formation that overlies the cave limits vadose water from entering the system [14], [17], [19]. The deep recesses of Lechuguilla Cave, isolated from surface input for the past 4–7 million years, therefore provide a unique environment to study the presence and prevalence of antibiotic resistance elements.

We report here a sampling of the culturable Lechuguilla Cave resistome – the antibiotic resistance genes found in a culture collection of isolates from this cave environment. In addition to finding a wide array of resistance in this culture collection, we also describe a novel macrolide kinase variant and an inducible daptomycin hydrolase, demonstrating not only that resistance is prevalent in the absence of anthropogenic antibiotics, but hitherto undiscovered mechanisms of resistance that have potentially important clinical implications are prevalent in the environment.

## Results

### Sampling of the Lechuguilla Cave Culturable Microbiome

Since its discovery in 1986, Lechuguilla Cave has been closed to human access without a permit. The sample areas were chosen off the designated trail through the cave, in areas that have experienced very little human impact (National Park records suggest that a maximum of 4–6 people have been in the vicinity of each sample site) (Figure 1). Specific sample locations were chosen due to the unlikelihood that these areas were actually exposed to human visitation: absence of footprints and scuffmarks that usually indicate human activity. Three sample sites were chosen, based on location and geology: LCDS1 and LCEAE (deep and within the Capitan Formation) and LCEA1 (mid-depth in the Yates Formation). None of the sites received any direct source of water with any moisture likely to come via percolation from the surface or from condensation events within the cave atmosphere. LCDS1 was the deepest sample site (at −400 m) within a region known as Deep Secrets and LCEAE was in the proximity of the LCDS1 sample site at the same depth. LCEA1 was chosen as an area that has been closed to human access for the past 20 years, with the only human activity in the area being carried out by scientists. The LCEA1 sample is at the bottom of the Yates Formation, which is high in iron and manganese and is the rock unit in which much of the oil and gas is extracted from the Delaware Basin. As a result, this rock unit is usually associated with organic material from ancient sediments [14].

A total of 20 different culture media were inoculated at each sample site, resulting in the isolation of over 500 unique isolates (Text S1) [20]. Of these, 93 that were able to grow in 50% TSB were chosen at random to screen for antibiotic resistance in this fraction of the cave microbiome (Table S1, Figure S1). These bacteria were phylogenetically classified by sequencing of the 16 S rRNA gene sequence (Table S2) and spanned several Gram-positive and Gram-negatives genera known to be associated with cave environments [21], [22], [23], [24], [25], [26], [27].

### Antibiotic Resistance Screen of the Lechuguilla Microbiome

**Figure 2:**
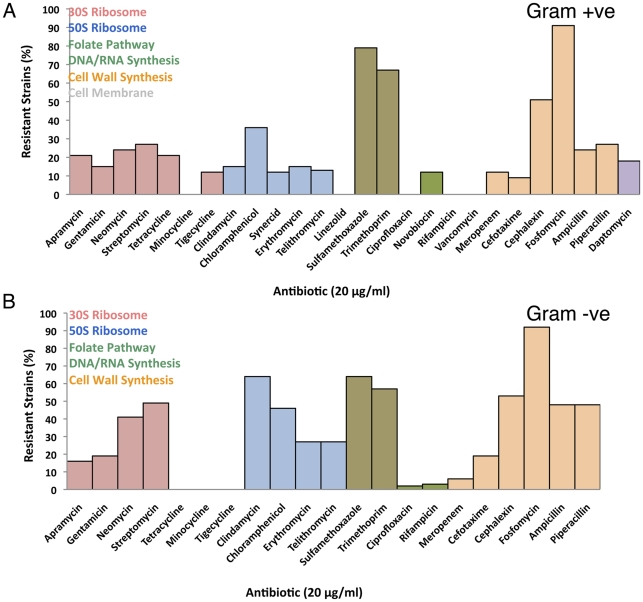
Resistance levels of Lechuguilla cave bacteria at 20 µg/ml against various antibiotics: (top) Gram-positive strains (bottom) Gram-negative strains. Antibiotics are grouped according to their mode of action/target, where each color represents a different target.

**Figure 3:**
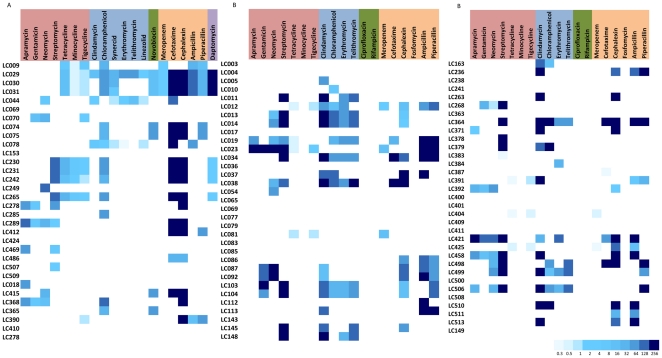
MIC of antibiotics determined in this study. Heat Plot for (A) Gram-positive strains (B) Gram-negative strains against various antibiotics. Antibiotics are grouped according to their mode of action and the gradient from light blue to dark blue represents the range from lowest MIC value (0.3 µg/ml) to highest MIC value (256 µg/ml) as shown in the legend. White means no MIC was determined.

The Lechuguilla bacterial collection (93 strains, 33% Gram-positive and 63% Gram-negative) was screened against 26 different antimicrobial agents representing a broad spectrum of natural products, their semi-synthetic derivatives, and completely synthetic molecules (Figure 2). In the primary screen, the level of resistance was assessed by monitoring the growth at 20 µg/ml drug concentration (a high level of antibiotic to select for robust resistance) and resistance in the primary screen was defined as >50% growth in the presence of antibiotic, consistent with other surveys of resistance [11], [28]. Resistant and sensitive strains obtained from the primary screen were further quantitatively analyzed by determination of the minimal inhibitory concentration (MIC) of the antibiotics (Figure 3).

Resistance spanned most of the major drug families in both Gram-positive and Gram-negative strains. In the collection of 33 Gram-positive strains, on average, 70% of the strains were resistant to 3–4 different antibiotic classes. Three strains were resistant to 14 antibiotics, all of which were *Streptomyces* spp. These values are consistent with those found in surface *Streptomyces* [11]; however, unlike surface strains, we saw no Gram-positive resistance to the synthetic drugs ciprofloxacin and linezolid, the semi synthetic compounds rifampicin and minocycline, and the natural product vancomycin (Figures 2–3).

Gram-negative bacteria are intrinsically resistant to many classes of antibiotics due to the presence of the relatively impermeant outer membrane along with the presence of chemo-selective porins and highly efficient small molecule efflux pumps. Therefore, only antibiotics known to have activity against Gram-negatives were included in the data analysis. On average, approximately 65% of the Gram-negative strains showed resistance to 3–4 antibiotic classes. No tetracycline resistance was observed, though it is common in surface bacteria [29], [30], [31]. On the other hand, resistance to sulfamethoxazole, trimethoprim, and fosfomycin was common; a feature shared with surface bacteria (Figures 2–3).

### Antibiotic Inactivation by Gram-positive isolates

Enzymes that modify antibiotics are of particular interest as they most likely evolved in direct response to the emergence of specific antibiotics to block their activity in contrast to mechanisms such as efflux that often can target several classes of bioactive compounds of diverse chemical structure [32]. Within the isolates, we detected no inactivation of aminoglycosides, the lincosamide clindamycin, or chloramphenicol; antibiotics where enzymatic inactivation is a prevalent resistance mechanism in surface bacteria (Table 1) [33]. On the other hand, substantial inactivation (22–62% of strains) was seen for ß-lactams (the penicillins ampicillin, piperacillin and the cephalosporin cephalexin), which is primarily caused by the hydrolysis of ß-lactam ring by ß-lactamases (Table 1) [34].

**Table 1:** Summary of Antibiotic Inactivation Studies for Gram-positive Isolates.

Antibiotic	Resistant Strains	Number of antibiotic Inactivation Strains	Mechanism of Inactivation
Apramycin	7	0	-------
Gentamicin	5	0	-------
Neomycin	8	0	-------
Streptomycin	8	0	-------
Tetracycline	4	0	-------
Minocycline	1	0	-------
Clindamycin	6	0	-------
Chloramphenicol	12	0	-------
Synercid	2	0	-------
Erythromycin	7	1	Phosphorylation
Telithromycin	4	4	Phosphorylation Glycosylation
Linezolid	2	0	-------
Novobiocin	5	0	-------
Cephalexin	17	5	Hydrolysis
Ampicillin	8	5	Hydrolysis
Piperacillin	9	2	Hydrolysis
Daptomycin	24	7	Hydrolysis

Strains were grown in 50% TSB for 5 days in presence of 20 µg/ml antibiotic. Conditional media was used for setting up disk diffusion assays and LC-MS analyses. Inactivation was defined as the absence of a zone of clearance around the disk. Hydrolytic mechanism of ß-lactam resistance is inferred.

**Figure 4:**
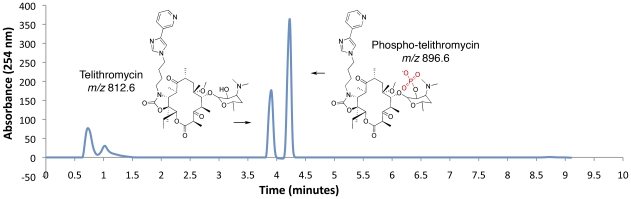
Inactivation of Telithromycin by *B. paraconglomeratum* strain LC44. After incubation of *B. paraconglomeratum* in the presence of 20 µg/ml telithromycin, chemical modification was determined by the loss of antimicrobial activity of the clarified culture supernatant associated with modification of telithromycin as assessed by LC-MS. LC-MS analysis of the resulting inactive extract indicated a shift of retention time and an increase in *m/z* by 80 consistent with phosphorylation.

Enzymatic inactivation was detected for macrolide antibiotics in four strains: three isolates of *Streptomyces* spp. and one of *Brachybacterium paraconglomeratum* (Table S3). These strains inactivated both the natural product erythromycin and its 3^rd^ generation semi-synthetic derivative telithromycin. Inactivation by the *Streptomyces* isolates yielded a product with a mass increase of 162, consistent with mono-glycosylation of the antibiotic [11], [35]. Inactive macrolide antibiotics from *B. paraconglomeratum* extracts on the other hand revealed a mass increase of 80, indicative of inactivation by phosphorylation (Figure 4) [36].

**Figure 5:**
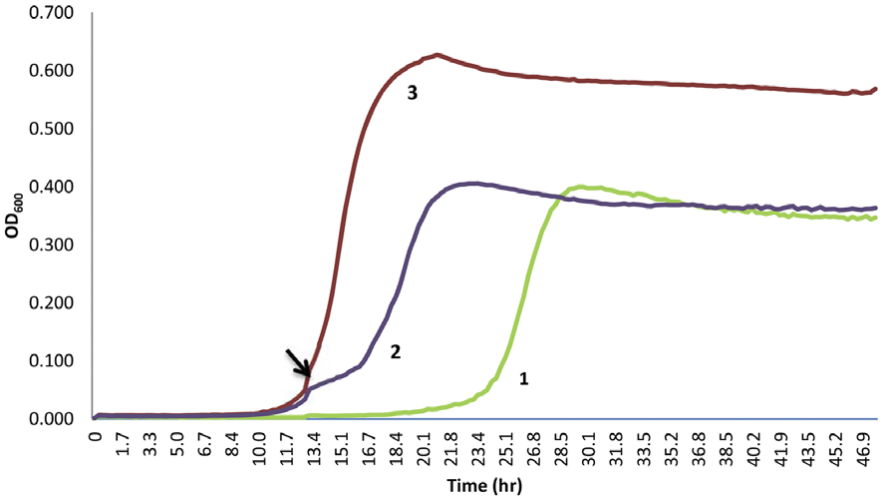
Induction of daptomycin resistance in *Paenibacillus lautus* LC231. LC231 was cultured in TSB supplemented with 1.25 mM CaCl_2_ and 4 µg/ml daptomycin added from start (zero time point) (1) or early log phase (2). Growth was compared to growth control with no drug (3). Arrow represents the time point at which daptomycin was added during early log phase.

The lipopeptide daptomycin is the newest class of antibiotic approved for clinical use. Three *Streptomyces* strains were found to be highly resistant to daptomycin (MIC≥256 µg/ml) and inactivated the antibiotic through hydrolysis as assessed by LC/MS (*m/z* increase of 18 g/mol). This result is consistent with the high level of daptomycin inactivation in surface *Streptomyces* where this is a constitutively expressed activity [11], [37]. Additionally, four isolates of *Paenibacillus lautus* completely inactivated daptomycin when grown at sub-MIC concentration of the antibiotic. Daptomycin inactivation has not been previously reported in low G+C bacteria (*Firmicutes* bacteria). Purification of the inactive product followed by MS/MS analysis revealed hydrolytic cleavage of the ester bond between the threonine and kynurenine residues resulting in ring-opening inactivation (Table S4). The inactivation of daptomycin was highly sensitive to inhibition by EDTA, but not significantly by Ser esterase/protease inhibitors (Figure S2), consistent with the possible involvement of a metallo-esterase. This contrasts with our recent analysis of *Streptomyces* daptomycin esterases, which appear to use canonical Ser catalytic triad chemistry to inactivate the antibiotic [37]. Furthermore, unlike inactivation by *Streptomyces*, which appears to be constitutively expressed, the production of the inactivating activity in *P. lautus* was inducible by exposure to daptomycin (Figure 5). To explore if this activity was unique to the cave isolate, we obtained a surface strain of *P. lautus* (ATCC 43898) and observed similar results in enzymatic inactivation and antibiotic-associated induction of activity.

### Antibiotic Inactivation by Gram-negative isolates

We observed little enzyme-mediated antibiotic inactivation in Gram-negative isolates (Table 2), suggesting that there are other molecular mechanisms of resistance at play such as efflux, target modification, or barriers to entry. Three strains belonging to the genera *Agrobacterium* and *Ochoromobactrum* displayed chloramphenicol inactivation, which we determined by LC/MS to be the modification by acetylation (Table S5); a well-established resistance mechanism both in Gram-positive and Gram-negative bacteria [33].

**Table 2:** Summary of Antibiotic Inactivation Studies for Gram-Negative Isolates.

Antibiotic	Resistant Strains	Number of antibiotic Inactivation Strains	Mechanism of Inactivation
Apramycin	10	0	-------
Gentamicin	11	0	-------
Neomycin	24	0	-------
Streptomycin	30	0	-------
Telithromycin	17	0	-------
Clindamycin	39	0	-------
Chloramphenicol	28	2	Acetylation
Trimethoprim	35	0	-------
Cephalexin	36	14	Hydrolysis
Cefotaxime	12	4	Hydrolysis
Ampicillin	30	13	Hydrolysis
Piperacillin	30	16	Hydrolysis

Strains were grown in 50% TSB for 5 days in presence of 20 µg/ml antibiotic. Conditional media was used for setting up disk diffusion assays and LC-MS analyses. Inactivation was defined as the absence of a zone of clearance around the disk. Hydrolytic mechanism of ß-lactam resistance is inferred.

### Characterization of a *B. paraconglomeratum* macrolide kinase

**Figure 6:**
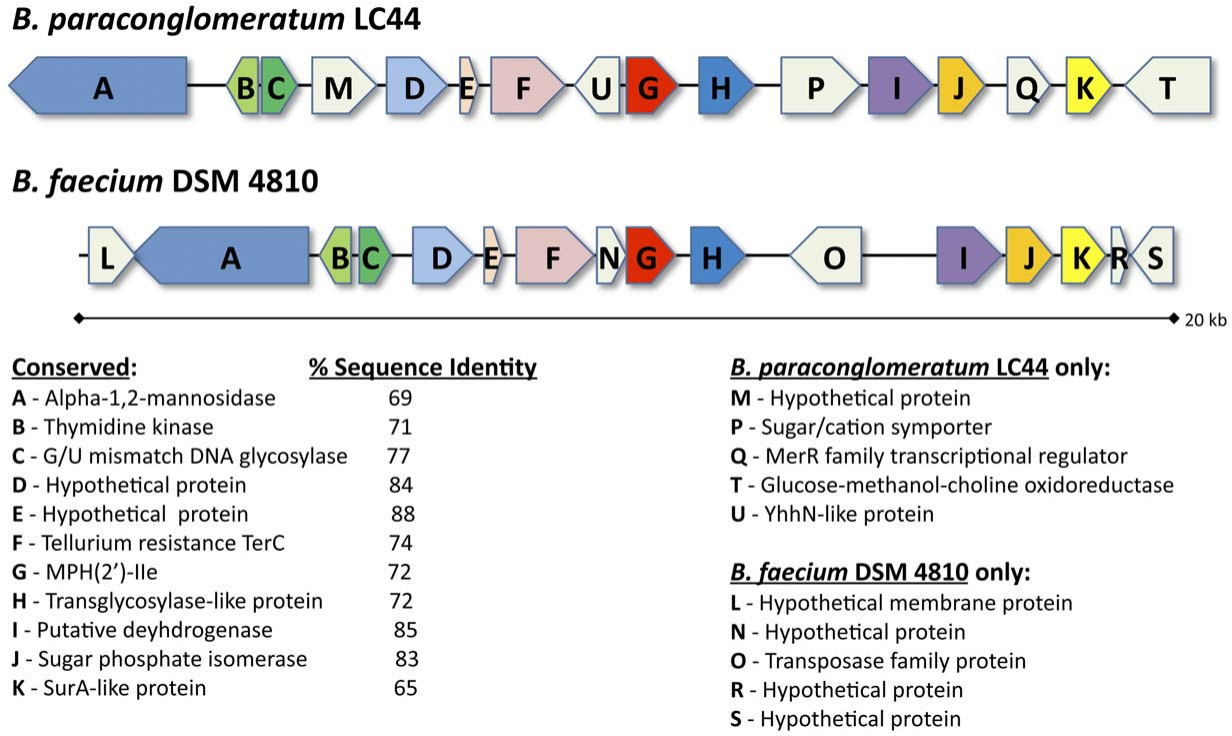
Genetic context of *mph* genes in *Brachybacterium* strains. A genetic map was constructed using available genome sequences of *Brachybacterium* strains and shown above is a schematic of translated protein query based on BLAST analysis. The MPH sequence is shown in red and homologous sequences are marked with identical colors.

Phosphorylation of macrolides in pathogenic bacteria is a growing clinical problem [38] catalyzed by a family of macrolide phosphotransferases (MPHs) [36], [39]. In order to probe for the presence of possible *mph* genes in macrolide inactivating *B. paraconglomeratum*, we prepared a draft genome sequence using Roche 454 and Illumina platforms. A gene encoding a predicted macrolide kinase, *mphE*, was identified (Figure 6). We expressed the gene product in *E. coli*, purified the enzyme and determined its activity and specificity using steady state kinetics (Table 3). The enzyme efficiently modified 14-, 15-, and 16-membered macrolide antibiotics aligning this enzyme with known type II MPHs (based on previous characterization of these enzymes in *E. coli* isolates) [40], [41]. The regiospecificity of phosphorylation of telithromycin was determined by multidimensional and multinuclear magnetic resonance analysis to be at the 2′-hydroxyl group of the desosamine sugar (Figures S3 and S4, Tables S6 and S7).

**Table 3:** Kinetic parameters for MPH (2′)-II from *B. paraconglomeratum.*

Substrate	Lactone Ring	K_m_ (µM)	*k_cat_* (s^−1^)	K_i_ (µM)	*k_cat_*/K_m_(s^−1^ M^−1^)
Erythromycin^1^	14	43.8±11.3	0.101	-	2.30×10^3^
Clarithromycin	14	21.9±5.45	0.106	-	4.82×10^3^
Telithromycin	14	12.2±5	0.04	600±285	3.52×10^3^
Roxithromycin	14	58.98±10.9	0.202	-	3.42×10^3^
Azithromycin	15	53.97±7.59	0.168	-	3.14×10^3^
Spiramycin	16	45.6±14.9	0.173	292±108	3.79×10^3^
Tylosin	16	222±29.2	0.197	66.4±8.92	8.87×10^2^
GTP^2^	-	10.2±0.53	0.093	-	9.3×10^4^

1- GTP held at 200 µM for antibiotic substrates, 2-erythomycin held at 400 µM.

The genome of another *Brachybacterium* species has been reported, *Brachybacterium faecium* DSM 4810, a terrestrial soil isolate [42]. The *B. faecium* genome includes a putatively annotated aminoglycoside kinase, that is 72% identical at the amino acid level to the *B. paraconglomeratum* MPH. Expression, purification and analysis of the *B. faecium* enzyme revealed similar antibiotic substrate profiles and activity as the MPH encoded by the *mphE gene in B. paraconglomeratum*, with no aminoglycoside modification activity (Table 4).

**Table 4:** Kinetic parameters for MPH (2′)-II from *B. faecium* DSM 4810.

Substrate	Lactone Ring	K_m_ (µM)	*k_cat_* (s^−1^)	K_i_ (µM)	*k_cat_*/K_m_(s^−1^ M^−1^)
Erythromycin^1^	14	38.2±6.24	0.192	-	5.03×10^3^
Clarithromycin	14	42.0±6.26	0.286	-	6.81×10^3^
Telithromycin	14	23.9±4.22	0.168	-	7.05×10^3^
Roxithromycin	14	19.3±2.60	0.262	-	1.36×10^4^
Azithromycin	15	60.6±8.86	0.312	-	5.18×10^3^
Spiramycin	16	17.3±3.18	0.157	-	9.10×10^3^
Tylosin	16	14.5±4.00	0.088	-	6.07×10^2^
GTP^2^	-	16.0±1.3	0.203	-	1.27×10^4^

1- GTP held at 200 µM for antibiotic substrates, 2-erythomycin held at 400 µM.

The *mph* genes in both *Brachybacterium* species are organized on comparable regions of the chromosome and flanked by similar genes and synteny (Figure 6); a survey 10 kb upstream and downstream of *mph* revealed eleven common and five different genes (Figure 6) and the gene products were 75–80% similar. Phylogenetic analysis shows that MPHs from *Brachybacterium* strains cluster together as a separate group among the known and putative members of the MPH family (Figure S5).

## Discussion

Antibiotic resistance is manifested through a number of different mechanisms including target alteration, control of drug influx and efflux, and through highly efficient enzyme-mediated inactivation. Resistance can emerge relatively quickly in the case of some mutations in target genes and there is evidence that antibiotics themselves can promote such mutations [43], [44], [45], [46]; however, resistance to most antibiotics occurs through the aegis of extremely efficient enzymes, efflux proteins and other transport systems that often are highly specialized towards specific antibiotic molecules. Such elements are the result of evolution through natural selection; this therefore implies that antibiotic resistance has a long evolutionary past. A growing body of evidence suggests that non-pathogenic environmental organisms are a reservoir of resistance genes that have the potential to be transferred to pathogens [31], [47], [48]. The problem of antibiotic resistance in clinical settings therefore likely has its origins in the environment.

One of the challenges in studying the evolution and prevalence of resistance is the massive use of antibiotics in the clinic and in agriculture over the past seven decades that makes identifying environments that have not been impacted by anthropogenic antibiotics difficult. Studying resistance in pristine environments that have not been exposed to human antibiotic use provides a critical measure of the genetic diversity of resistance that is essential to our understanding of resistance gene prevalence and evolution. Lechuguilla Cave provides an outstanding ecosystem that has been isolated for over 4 million years. The cave's geologic features, including the impermeable siltstone caprock which prevents rapid influx of surface water, great depth, and long isolation from the surface, rules out the possibility of exposure to anthropogenic use of antibiotics as well as antibiotic contamination through water bodies. As a result Lechuguilla Cave is an ideal ecosystem for investigating microbes that have not been exposed to anthropogenic antibiotics.

We surveyed the antibiotic susceptibility of 93 bacterial strains isolated from Lechuguilla Cave. This was a genetically diverse collection of oligotrophic organisms (Figure S1), highly adapted to survive in a nutrient limited environment [22]. Like surface organisms [11], the majority of these strains were multidrug resistant indicating that antibiotic resistance is a common and widespread phenotype in pristine, unimpacted environments; however, there are differences in the pattern of resistance. For example, we measured little resistance to the synthetic antibiotics ciprofloxacin and linezolid, while resistance to natural product antibiotics was more prevalent. Unlike surface bacteria, we also detected very little resistance to tetracycline, glycopeptide (vancomycin), rifamycin (rifampicin) and lipopeptide (daptomycin) natural product antibiotics. There are several possible reasons for these differences. First, this survey includes multiple bacterial genera across five phyla, while our original sampling focused on actinomycetes [11]. As prodigious producers of natural products including antibiotics, it is logical that actinomycetes would also be enriched in resistance elements. Second, the isolate sample size is smaller in this study in comparison to our previous study and we have likely not examined the full resistome of both the culturable and non-culturable microbiome. Further studies of both surface and cave microbiomes including more extensive cultivation and metagenomic analysis are therfore necessary to interpret the results with more confidence.

Aminoglycoside antibiotic resistance was more common in Lechuguilla Cave isolates as compared to surface actinomycetes. This may reflect the biosynthetic capacity of antibiotic producing bacteria in Lechuguilla Cave and the production of these antibiotics by species within the cave. A survey of the actinomycetes in our collection using oligonucleotide primers designed to amplify aminoglycoside biosynthetic genes failed to identify potential aminoglycoside producers; however linking resistance to antibiotic production will require an extensive and systematic survey of the cave microbiome and resistome that is beyond the objectives of this work.

The mechanisms of antibiotic modification and inactivation are evidence of highly specific evolutionary adaptations to evade the cytotoxic action of these antibiotics. The high level of ß-lactam antibiotic resistance by hydrolysis parallels that of surface bacteria and the result of genetically diverse ß-lactamases that are widespread in microbial genomes. Similarly, chloramphenicol acetylation was also detected, an activity that is well established in surface bacterial isolates [49]. Nonetheless, the hydrolytic inactivation of daptomycin in isolates of *P. lautus* was unexpected. We recently showed that high G+C content actinomycetes use a hydrolytic ring-opening reaction as a common strategy of daptomycin inactivation [37], and this work further exposes the susceptibility of daptomycin's structure to hydrolytic cleavage. We also provide the first evidence that daptomycin inactivation can occur within the low G+C content bacteria *Firmicutes*, for which daptomycin's use is approved; a mechanism that clinical microbiologists should be on alert for emergence in pathogens. The inducible activity in *P. lautus* is very likely catalyzed by an EDTA-sensitive ring-opening esterase/protease [Figure S3]. Our efforts to purify the associated protein were unsuccessful due to instability of the activity (possibly by autocatalytic digestion); however, a similar inducible activity was recapitulated in a surface strain of *P. lautus*. This is intriguing as it suggests either involvement of a specific receptor for daptomycin or a non-specific response to the physiological impact of daptomycin bioactivity.

The observation of two distinct macrolide inactivation mechanisms in the Lechuguilla bacterial isolates was also intriguing. In the resistant *Streptomyces* strains we determined that antibiotic modification by glycosylation was the primary mechanism of inactivation, a mechanism that is known in surface actinomycetes [11]. On the other hand, we established that the mechanism of macrolide inactivation in *B. paraconglomeratum* is through phosphorylation at position 2′ catalyzed by a member of the MPH class of antibiotic kinases. Previously identified *mph* genes are encoded on plasmids found in clinically resistant isolates of the pathogens *Escherichia coli*, *Staphylococcus aureus*, *Pasteurella multocida* and *Pseudomonas aeruginosa* [36]. This is the first report of *mph* genes from environmental bacteria as a potential source of the genes currently circulating in pathogens. The presence of a transposase-like gene upstream of *mph* from a surface strain of *B. faecium* points to a potential history of horizontal gene transfer (Figure 6). It is possible that *mph* genes have been circulating among bacterial populations before the cave was sealed off millions of years ago, resulting in an *mph* gene that is a shared trait between both terrestrial and cave bacteria.

There are two likely explanations for retention of the *mph* genes with same biochemical properties despite the long isolation of the *Brachybacterium* strains: (i) these genes could serve a physiologic or metabolic function unrelated to antibiotic resistance (although the genetic context (Figure 6) does not suggest an obvious role); or (ii) these genes are resistance elements for conferring antibiotic resistance. We could not detect any macrolide biosynthetic gene clusters in bacteria collected in the same region as *B. paraconglomeratum* (as evidenced by a absence of the signature macrolide D-desosamine biosynthesis gene, *eryCVI*, not shown); however the actinomycete small sample size does not rule out the possibility of the presence of hitherto undetected macrolide producers within the cave.

This work demonstrates that antibiotic resistance is widespread in the environment even in the absence of anthropogenic antibiotic use. Lechuguilla Cave represents a remarkable ecosystem that has been isolated for millions of years, well before the clinical and agricultural use of antibiotics. The presence of multidrug resistant organisms even in this pristine environment reinforces the notion that the antibiotic resistome is an ancient and pervasive component of the microbial pangenome. Given the nutrient-limited nature of the cave environment, it is likely that competition for resources plays a dominant role in species persistence. This competition may occur through numerous adaptations, from changes in cell physiology and growth, to the production of antimicrobials to outcompete nutritional rivals. Equally important could be the acquisition and development of defense mechanisms to enable flexibility and growth in the presence of noxious bioactive compounds and limit the effectiveness of such competitors in the ecosystem.

Given the relatively small sample size of this study, the observation of two previously unidentified mechanisms of antibiotic resistance (e.g. daptomycin hydrolysis) suggests that significant genetic diversity may be present in the environment and capable of being marshaled in the presence of antimicrobial agents. The available genetic diversity extant in microbial genomes dwarfs our ability to introduce antibiotics in to clinical use. Furthermore, the microbial chemical ecology of bioactive compounds such as antibiotics is not well understood and as such the resistome includes mechanisms that very likely have not been evolved simply to evade the effects of molecules that we have termed antibiotics [50]. This fact further underlines the importance of the judicious use of antibiotics to avoid selection of existing resistance elements and their subsequent mobilization through microbial communities thereby limiting the effectiveness of these drugs to treat infectious diseases. The remarkable genetic diversity of the antibiotic resistome, uncovered in this and other studies has additional practical application as an ‘early warning system’ for new drugs introduced into the clinic. Resistance mechanisms in the environmental resistome can emerge in the clinics and the clinical community should be aware of them; for example our discovery of hydrolytic mechanisms of daptomycin resistance. Finally, the diversity in the resistome also suggests that there are a myriad of bioactive molecules with antibiotic properties waiting to be discovered. Some of these may have the potential to be productive leads as antibiotics or as improved scaffolds that can evade existing clinical resistance.

## Materials and Methods

### Antibiotic Resistance Screen of Cave Strains

A sample of 93 bacterial strains were isolated from three deep, remote sample sites in Lechuguilla Cave (Figure 1) under a permit for sample collection provided by the US National Park Service (Permit CAVE-2007-SCI-0009 for Study CAVE-00049). These strains were isolated on a variety of culture media that resembled the carbon and energy sources thought to be available to microorganisms within the cave (see Text S1). Following single colony isolation, these isolates were grown in dilute (50%) Tryptic Soy Broth (TSB) at 30°C for 3–5 days and frozen stocks were prepared in 96 well plate format (1∶10 dilution of cultures in 80% glycerol). Master plates containing 150 µl 50% TSB were inoculated from the frozen stock plates using a replica head platter and grown at 30°C for 3–5 days. The inoculum plate was prepared from the master plate as a 1∶100 dilution in 50% TSB. Assay plates containing 50% TSB (140 µl volume) in the presence and absence of 20 µg/ml antibiotic (named resistance plates and growth plates respectively) were inoculated with 10 µl/well of the inoculum plate and incubated at 30°C for 5 days. All studies using the antibiotic daptomycin were carried out in cultures supplemented with 1.25 mM–2.5 mM CaCl_2_. Screens were performed in duplicate and data collected as OD_600_ after 5 days. Resistance plate data were normalized to the growth control plates and resistance was defined as more than 50% growth in the presence of an antibiotic.

### Secondary assays of resistance

Strains positive for antibiotic resistance in the primary screen were confirmed by determination of their MIC and further analyzed for their ability to inactivate the antibiotic. To determine MIC, cultures (5 ml) containing 50% TSB were inoculated with a single isolated colony of each strain of interest and grown shaking at 250 rpm at 30°C for 5 days. MIC determinations were conducted in 96 well U- bottom MIC plates (VWR) containing 50% TSB media and supplemented with antibiotic, (ten 2-fold serial dilution of antibiotic, final concentration ranging from 0.5 µg/ml to 128 µg/ml). Cultures were diluted to OD_600_ of 0.08–0.1 in 0.85% NaCl and MIC plates were inoculated with 1∶20 dilution of this suspension. Plates were incubated at 30°C for 5 days. *Escherichia coli* ATCC 29522, *Staphylococcus aureus* ATCC 29213 and *Enterococcus faecalis* ATCC 29212 were used as control strains. MIC testing was performed in duplicate, where MIC was defined as the drug concentration showing no visible growth.

In order to evaluate resistant strains for antibiotic inactivation, cultures containing 5 ml of 50% TSB in the presence and absence of 20 µg/ml of antibiotic were inoculated with 3–5 single isolated colonies of strain of interest and cultures were grown at 30°C for 5 days. Uninoculated controls were also prepared. After growth at 30°C for 5 days, cultures were centrifuged for 20 minutes at 16,800×*g* and conditioned media was collected. One of the following susceptible organisms/test organisms was used for antimicrobial susceptibility testing (antimicrobial disk diffusion assay): *Bacillus subtilis*, *Micrococcus luteus* and *Staphylococcus saprophyticus* ATCC 15305. Inocula of test organisms were prepared to the 0.5 McFarland standard using the direct colony suspension method according to the Clinical and Laboratory Standard Institute guidelines [51]. Susceptible organisms were plated on LB Agar and conditioned media (20–30 µl) was spotted on sterile paper disks prior to incubation at 37°C overnight. Inactivation was defined as complete absence of a zone of inhibition.

### *B. paraconglomeratum* LC44 Genome Sequencing and Assembly

*B. paraconglomeratum* LC44 was grown in 5 ml 50% TSB at 30°C for 5 days. Genomic DNA was isolated using QIAGEN DNeasy Blood and Tissue Kit 250 (Qiagen, Germany) with one modification of the manufacturer's protocol for cell lysis: 4 µl of RNase (100 mg/ml in Buffer TE) was added to the reaction mixture and incubated at room temperature for 2 minutes before washing the spin column. Genomic DNA was submitted for shotgun sequencing to Roche 454 Life Sciences Genome Sequencer at Farncombe Metagenomics Facility, McMaster University. Approximately one quarter of a 454 PTP was used for Titanium pyrosequencing on a 454 GS FLX. Additionally, 8059104 Illumina GAIIx 71 bp paired end reads were sequenced by Ambry Genetics (Aliso Viejo, California). After aggressive quality trimming and filtering, the approximately 5.5 million remaining Illumina reads (about 70× coverage) and 262995 454 reads (about 23× coverage) were assembled using MIRA version 3.4 with the ‘-job = denovo,genome,454,accurate,solexa’ switches [52]. The resulting contig sequences of the assembly were deposited in Genbank as a WGS project under accession AGSO00000000. The protein sequence of *E. coli* MPH (2′)-Ia (accession BAA03776) was used to query the assembled sequences using translated blast in order to find a gene responsible for the observed macrolide phosphotransferase activity. A good candidate was found on contig AGSO00000004 beginning at bp 76533 and a blastx search was performed against the NCBI non-redundant protein database where the top hits corresponded to sequences annotated as putative APH and MPH proteins.

### Cloning, Expression and Purification of Macrolide Phosphotransferases

The candidate macrolide phosphotransferase from *B. paraconglomeratum* (*mphE*) was synthesized by GenScript (USA) with codon optimization for *E. coli* expression and cloned into pET28b with *Nde* I and *Hind* III restriction sites. The plasmid containing MPH was transformed into *E. coli* BL21 (DE3) and the resulting colonies were grown overnight at 37°C in LB media supplemented with 50 µg/ml kanamycin. For overexpression, 1 L LB cultures supplemented with 50 µg/ml kanamycin were subcultured from the overnight culture at 1% (v/v) and grown at 37°C to an OD_600_ of 0.5–0.7. The cells were chilled on ice for 15 minutes. Protein expression was induced with 1 mM isopropyl-β-D-thiogalactopyranoside at 16°C for 16 hours. The cells were harvested and stored at −20°C until further use. For protein purification, the cells were resuspended in lysis buffer (50 mM HEPES, 300 mM NaCl, 10 mM Imidazole, pH 7.5), 1 mM phenylmethanesulfonylfluoride and 1 µg/ml pancreatic-bovine DNase and cells were lysed using T-S series cell disrupter. Protein was purified by standard immobilized metal affinity chromatography followed by gel filtration and stored in 10% glycerol at −20°C.

### Steady State Kinetic Analysis of MPH(2′)

Kinetic parameters of MPHs were determined in triplicate using Pyruvate Kinase/Lactate Dehydrogenase coupled assay [53]. Phosphorylation of macrolides by MPHs was monitored by absorbance of NADH (340 nm) in 96 well format using a SpectraMax reader. The reaction was initiated with macrolide or nucleotide (GTP). When monitoring macrolide dependence, 200 µM GTP was used and the macrolide concentration ranged from 3.2 µM and 400 µM. For nucleotide dependence, 200 µM macrolide was used and the final concentrations of GTP ranged from 7.82 µM to 2000 µM. All reactions were performed in triplicate.

The initial rates were fit to equation 1 or 2 (substrate inhibition) using Grafit 4.0 software (Erithacus Software, Staines, UK):
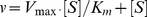
(1)

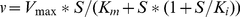
(2)


### Growth Analysis of *Paenibacillus lautus* LC231 with Daptomycin

Analyses were performed in 96-well flat bottom plates (200 µl volume total) using 50% TSB supplemented with 1.25 mM CaCl_2_ as a growth medium. Inocula represented 1∶200 dilutions of an overnight culture, standardized to an OD_600_ of 0.1. The following conditions were tested in duplicate: (**1**) no daptomycin, (**2**) 4 µg/ml daptomycin and (**3**) 4 µg/ml daptomycin added after 10.5 hours (early log phase). Plates were incubated while shaking at 30°C and the data was collected as an OD_600_ every 30 minutes using a Tecan Sunrise plate reader. Similar studies were conducted with a surface strain of *P. lautus* (ATCC 43898) except that the organism was grown in full strength TSB.

## Supporting Information

Text S1 **Supporting materials and methods.**
(DOCX)

Figure S1 **Lechuguilla cave microbiome.** A) Chart of the distribution of bacterial isolates sampled in this work. B) Consensus phylogram of bacterial strains used in this study. The tree was created using 16 S rRNA gene sequences. Node values represent the likelihood of the represented partition at each branch (based on 1000 maximum likelihood bootstrap analyses).
(TIF)

Figure S2 **Inhibition of daptomycin inactivation by a series of common protease and esterase inhibitors.** Inhibition studies were performed with crude culture supernatant in triplicate at 200 µg/ml of daptomycin with all traces of calcium previously removed. Error bars are 1 S.D..
(TIF)

Figure S3 **Structure of telithromycin and telithromycin phosphate.**
(TIF)

Figure S4 **^31^P – NMR spectra of telithromycin phosphate.**
(TIF)

Figure S5 **Phylogenetic Analysis of MPH/MPH-like proteins.** Aminoglycoside Phosphotransferases (APHs) were collapsed into a single branch (APH sequences) which was used as an outgroup in this analysis of amino acid sequence. Note that the scale bar represents 0.1 mutations/site.
(TIF)

Table S1 **Media Used for Initial Bacterial Cultivation.**
(DOCX)

Table S2 **List of strains used in this study.**
(DOCX)

Table S3 **Telithromycin Inactivation in Cave Isolates.**
(DOCX)

Table S4 **Tandem mass spectrometry analysis of ring-opened daptomycin by** ***Paenibacillus lautus*** **LC231.**
(DOCX)

Table S5 **Chloramphenicol Inactivation in Cave Strains.**
(DOCX)

Table S6 **^1^H Chemical Shifts of Telithromycin and Inactivated Product of** ***B. paraconglomeratum*** **LC44 in DMSO-d_6_ (ppm).**
(DOCX)

Table S7 **^13^C Chemical Shifts of Telithromycin and Inactivated Product of** ***B. paraconglomeratum*** **LC44 in DMSO-d_6_ (ppm).**
(DOCX)
