# Supplementary material for: Antibiotic Resistance Is Prevalent in an Isolated Cave Microbiome
Source: PLoS One. 2012 Apr 11;7(4):e34953. doi: 10.1371/journal.pone.0034953 (PMC3324550; doi:10.1371/journal.pone.0034953)
Supplement: Text S1 — Supporting materials and methods. (DOCX) [file pone.0034953.s001.docx]

**Text S1. SUPPLEMENTARY MATERIALS AND METHODS**

**Isolation of Cave Strains.** Unless stated otherwise, all chemicals were obtained from Sigma-Aldrich. Nine different types of media were used for cultivation, based on what we imagined to be potential carbon and energy sources available within the environment (Supplemental Table S1). To a subset of these media we added antibiotics (20 µg/ml tetracycline; 10 µg/ml nalidixic acid, 20 µg/ml chloramphenicol or 40 µg/ml ampicillin) to limit fast growing species such as *Pseudomonads,* which can rapidly overwhelm culture plates and make it difficult to isolate unique species. Water was collected from a pool within travel distance of each sample site and filter-sterilized using a 0.22 mm nitrocellulose filter. A Dremel tool was used to cut ~ 5 g piece of wall rock at each sample site, which was crushed *in situ* using a sterile steel pestle and mortar. Approximately 1 g of this material was then placed in 2 ml of sterile water and mixed by shaking. The course material was then allowed to precipitate out, and the supernatant was used to inoculate each of the prepared culture media. To prevent cross-contamination of carbon sources/nutrients, fresh swabs were used for each plate. Uninoculated plates of F, H, F/C and P/CAS media were used to confirm that any colonies observed were not contaminants (either through preparation or during sample collection). The cultures were then carried out of the cave and shipped to the laboratory at 4°C (2 days), before incubating in the dark at 21°C (the temperature of Lechuguilla Cave) for anywhere from 2 – 6 weeks. Unique colonies were then single colony isolated, initially on the same media on which they were isolated. After several passages on media, these strains were transferred to a 50% concentration of trypticase soy agar (TSA; (Difco™, Becton Dickinson, Franklin Lakes, NJ).

**DNA Extraction/16S Analysis of Cave Isolates.** To identify each isolate, the genomic DNA was extracted using a ZR Fungal/Bacterial DNA Kit (Zymo Research, Orange, CA) and the 16S ribosomal RNA gene sequence identified as previously described [1]. Sequences that were difficult to identify in this manner were cloned into a pTOPO-TA Cloning Vector (Invitrogen Corp., Carlsbad, CA) before sequencing. All sequencing was carried out at the University of Kentucky Advanced Genetic Technologies Center (<http://www.uky.edu/Centers/AGTC>) and deposited in the NCBI database under accession numbers JN863435-JN863529. DNA alignments were carried out using a NAST aligner [2] with manual corrections in the ARB Software Package (<http://mpi-bremen.de/molecol/arb>) (Fig. S6). For evolutionary distance calculations, a maximum likelihood algorithm using a general time reversal (GTR) substitution model was carried out in RAxML 7.2.7 with 1000 bootstrap replicates [3,4,5]. In all cases, *Anabaena oryzae* was used as the outgroup.

**LC-ESI-MS.** LC-ESI-MS analysis was performed on conditioned media to identify the inactivation products using an Agilent 1100 Series LC system and a QTRAP LC/MS/MS System (ABSciex). The reverse phase HPLC conditions are as follow: isocratic 5% solvent B (0.05% formic acid in acetonitrile), 95% solvent A (0.05% formic acid in water) over 1 min, followed by a linear gradient to 97% B over 7min at a flow rate of 1 ml/min and C_18_ column (Sunfire,5 μm, 4.6x50 mm).

**Phylogenetic Analysis of Macrolide Phosphotransferases.** We further investigated the relationship between known and putative MPH and APH sequences since they fall in a more broadly defined APH family. The putative LC44 MPH along with the top five hits of the previous blast search were added to a list of APH and MPH sequences previously analyzed [6]. The accessions of the additional sequences used are YP_003155631, ZP_05915402, ZP_05749839, NP_737713 and ABF87557 respectively. The sequences were aligned to the hidden Markov model for the APH family from PFAM release 4.1 (accession PF01636) using HMMER3 [7,8]. This alignment was refined using MUSCLE v3.7 [9] followed by manual inspection and small corrections to ensure that known phosphotransferase sequence motifs were successfully aligned. This alignment was used to estimate a phylogeny using MrBayes v3.1.2 [10]. Two runs of five million generations of MCMC using 4 chains were computed. A fixed WAG model was specified along with gamma distributed rate variation and a proportion of invariant sites. A rooted consensus tree was generated using the 50% majority rule from samples drawn every 100 generations using a burn-in of 1000 and specifying the APH sequences as an outgroup.

**Purification and characterization of Telithromycin Inactivation Product.** Large scale purification of phosphorylated telithromycin was performed for NMR analysis. Reaction containing 2.5 ml of buffer (500 mM HEPES, 400 mM KCl, 100 mM MgCl_2_ ), 200 µM GTP, and 3 ml of 2 mg/ml purified MPH(2’)-IIe from LC44 was initiated using 1 ml of 25 mM telithromycin. The reaction progress was followed to completion by LC-ESI-MS as described above. After lyophilization, anion exchange chromatography (Combiflash System, Teledyne Isco) on Redi*Sep*® Rf SAX column (5.7g, 40-63 µm, 60 angstroms) was used to purify the product. After washing the column with 90% Solvent A (dichloromethane) and 10% Solvent B (methanol), phosphorylated telithromycin was eluted using 1% ammonium hydroxide in methanol. Approximately 10 mg of pure phosphorylated telithromycin (> 98%) was obtained which was analyzed by multidimensional and multinuclear magnetic resonance analysis.

**Purification and Characterization of Inactivated Daptomycin.** A starter culture of *Paenibacillus lautus* LC231 was incubated overnight in 100% TSB at 30 °C and was used to inoculate 50 ml of M9 Minimal media (2% inoculum) containing 200 µg/ml of daptomycin. After incubating at 30 °C and 250 rpm for 3 days, cells were removed by centrifugation at 3,500 × *g,* and the supernatant was lyophilized. The resulting sample was resuspended in water and desalted using Sep-Pak® Plus C18 cartridge (Waters). After elution with 50% (vol/vol) acetonitrile, and concentration by lyophilization, the sample was further purified on a Sunfire ^TM^ Prep C18 OBO column (5 µm) and a Waters AutoPurification HPLC/MS. A linear gradient of dH_2_O and acetonitrile was used, both supplemented with 0.01% trifluoroacetic acid. Fractions with a mass-to-charge ratio (*m/z*) of 1640 ± 1 were collected, lyophilized and the resulting compound was dissolved in dH_2_O to a concentration of 5 mg/ml. The sample was analyzed by tandem mass spectrometry as previously described [11]. For sample preparation, inactivated daptomycin was dissolved in 5% acetonitrile/water to give a final concentration of 2 pmol/µL.

***In vitro* Inhibition Studies of Crude *Paenibacillus lautus* LC231 Supernatant.** A 25 ml of 50% TSB and 20 µg/ml daptomycin was inoculated with a single colony of *Paenibacillus lautus* LC231 and incubated at 30 °C and 250 rpm for 3 days. The culture was centrifuged at 10,000 × *g* for 10 minutes and the resulting supernatant was concentrated to a final volume of 1.5 ml using an Amicon Ultra-15 centrifugation filter (3000 MW cutoff, Millipore). Enzyme reactions, prepared in triplicate to a final volume of 250 µl contained the following: 50 µl of concentrated culture supernatant, 50 mM HEPES pH 7.5, 200 µg/ml daptomycin, and one of eight protease inhibitors. Inhibitors were used at the following concentrations: EDTA (50 µM), benzamidine (50 µM), 4-(2-Aminoethyl) benzenesulfonyl fluoride hydrochloride (AEBSF, 20 µM), pepstatin (10 nM), phenylmethanesulfonylfluoride (PMSF, 10 µM), phenanthroline (50 µM), leupeptin (1 µM) and chymostatin (1 µM). Reactions were performed for 6 hours at 30°C, and stopped with 50 µl methanol.

HPLC Analysis was performed using 2535 Quaternary pump system and 2998 Photodiode Array Detector (Waters). After injection on an XSelect CSH C18 column (Waters, 5 µm, 4.6 x 100 mm), separation was performed using an isocratic method of 37% acetonitrile supplemented with 0.1 % TFA. An injection volume of 20 µl was used. Peak areas for linearized daptomycin and intact daptomycin were determined, and activity was defined by the ratio of peak area for inactivated daptomycin to the combined total. Relative activities were determined by comparison to reactions devoid of inhibitors.
