# Supplementary figures and images for: Antibiotic Resistance Is Prevalent in an Isolated Cave Microbiome
Source: PLoS One. 2012 Apr 11;7(4):e34953. doi: 10.1371/journal.pone.0034953 (PMC3324550; doi:10.1371/journal.pone.0034953)

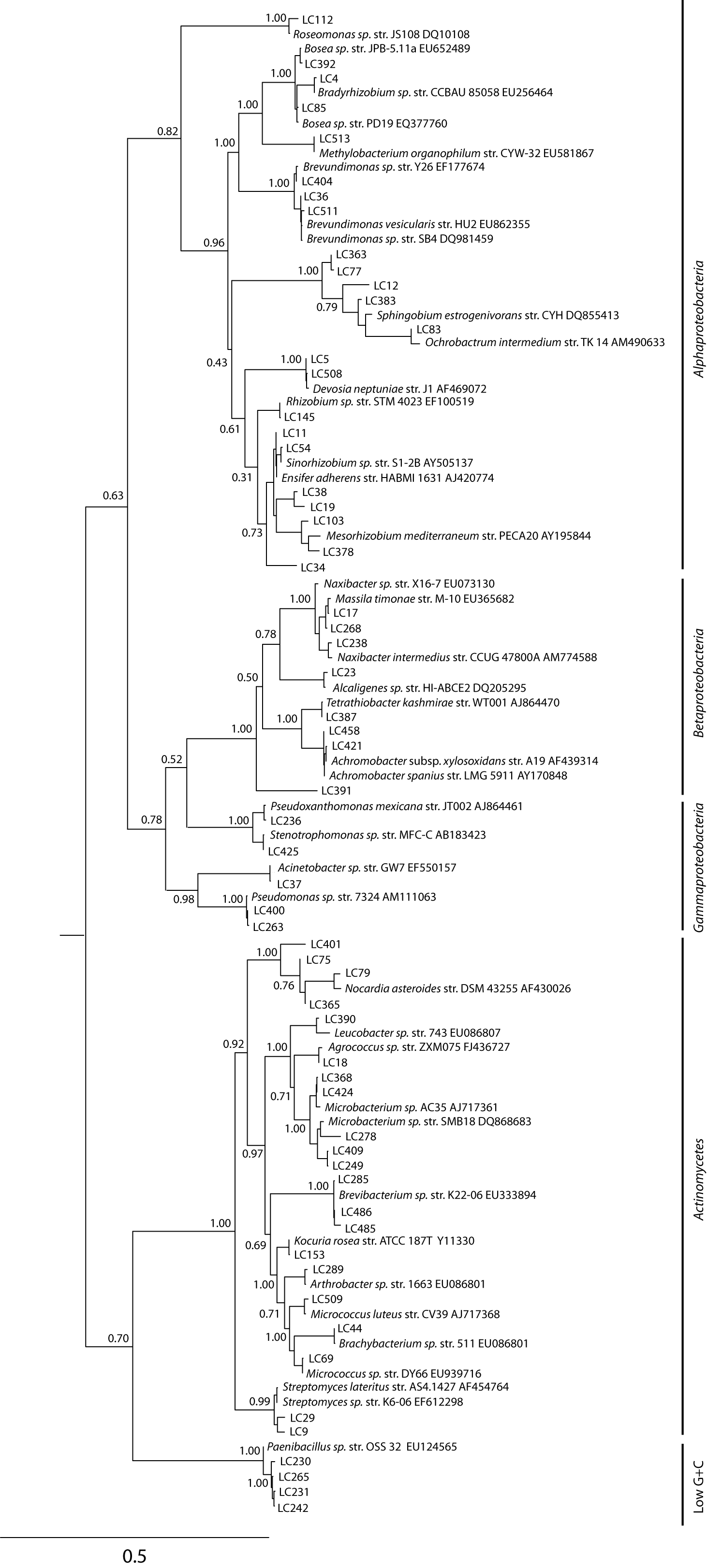

Supplement: Figure S1 — Lechuguilla cave microbiome. A) Chart of the distribution of bacterial isolates sampled in this work. B) Consensus phylogram of bacterial strains used in this study. The tree was created using 16 S rRNA gene sequences. Node values represent the likelihood of the represented partition at each branch (based on 1000 maximum likelihood bootstrap analyses). (TIF) [file pone.0034953.s002.tif]

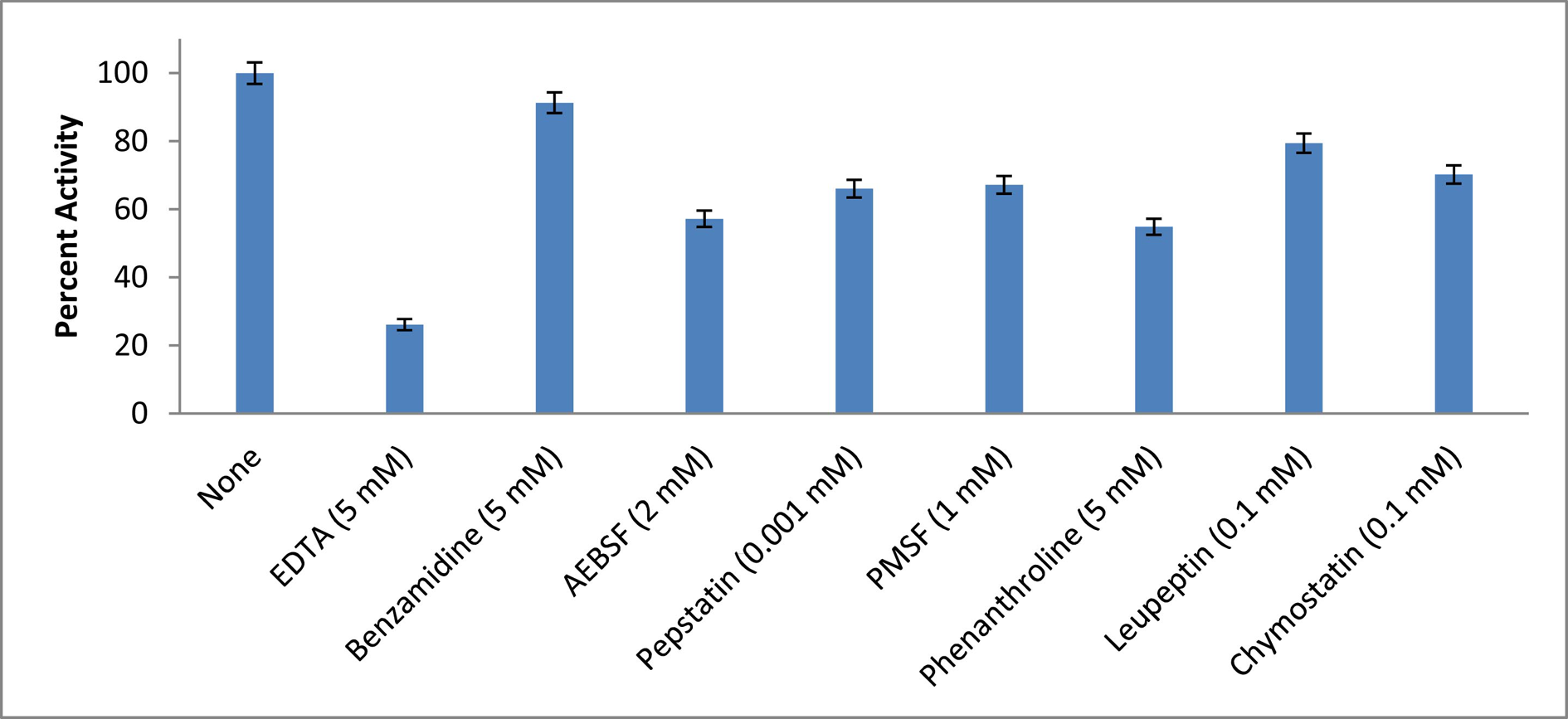

Supplement: Figure S2 — Inhibition of daptomycin inactivation by a series of common protease and esterase inhibitors. Inhibition studies were performed with crude culture supernatant in triplicate at 200 µg/ml of daptomycin with all traces of calcium previously removed. Error bars are 1 S.D.. (TIF) [file pone.0034953.s003.tif]

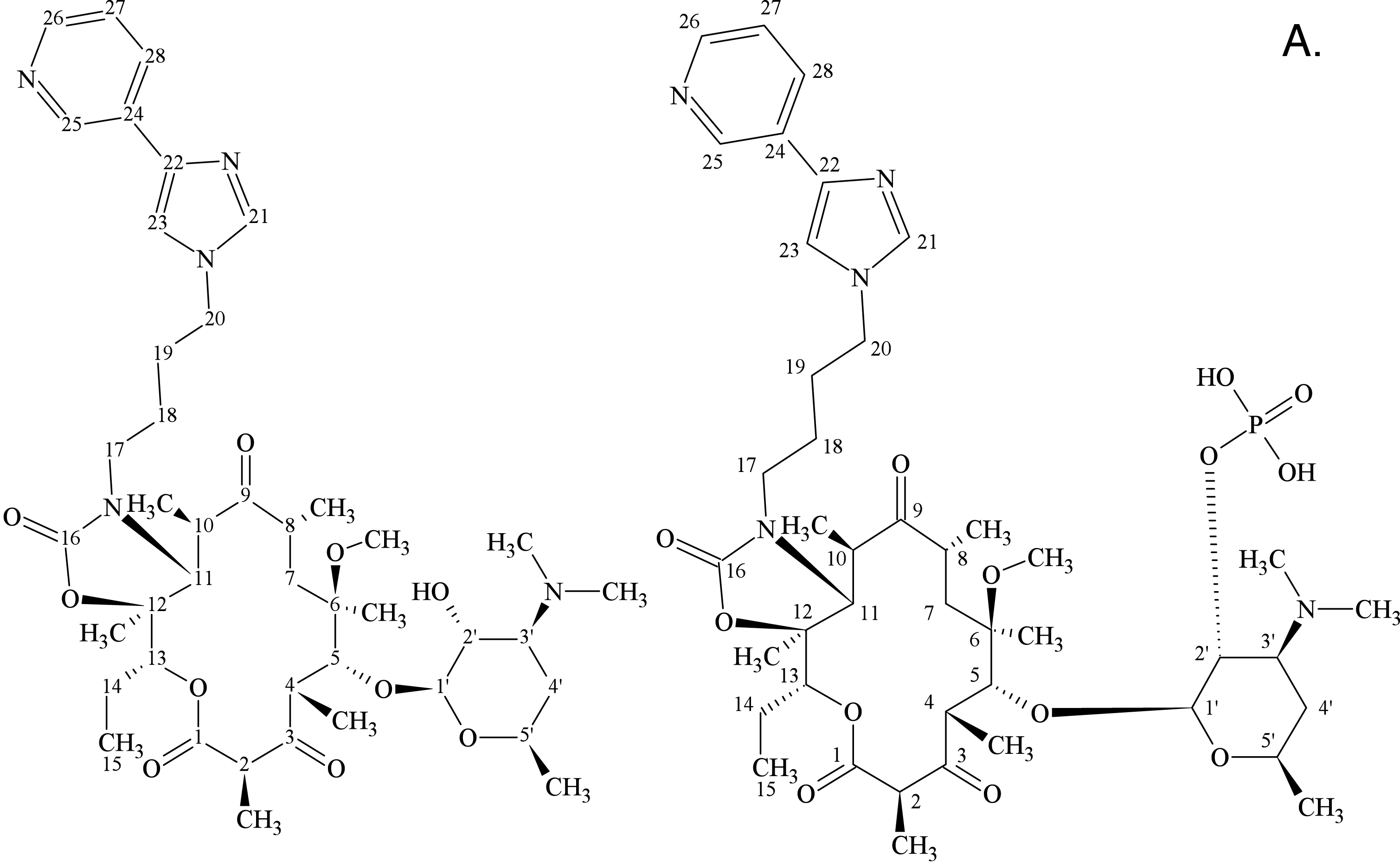

Supplement: Figure S3 — Structure of telithromycin and telithromycin phosphate. (TIF) [file pone.0034953.s004.tif]

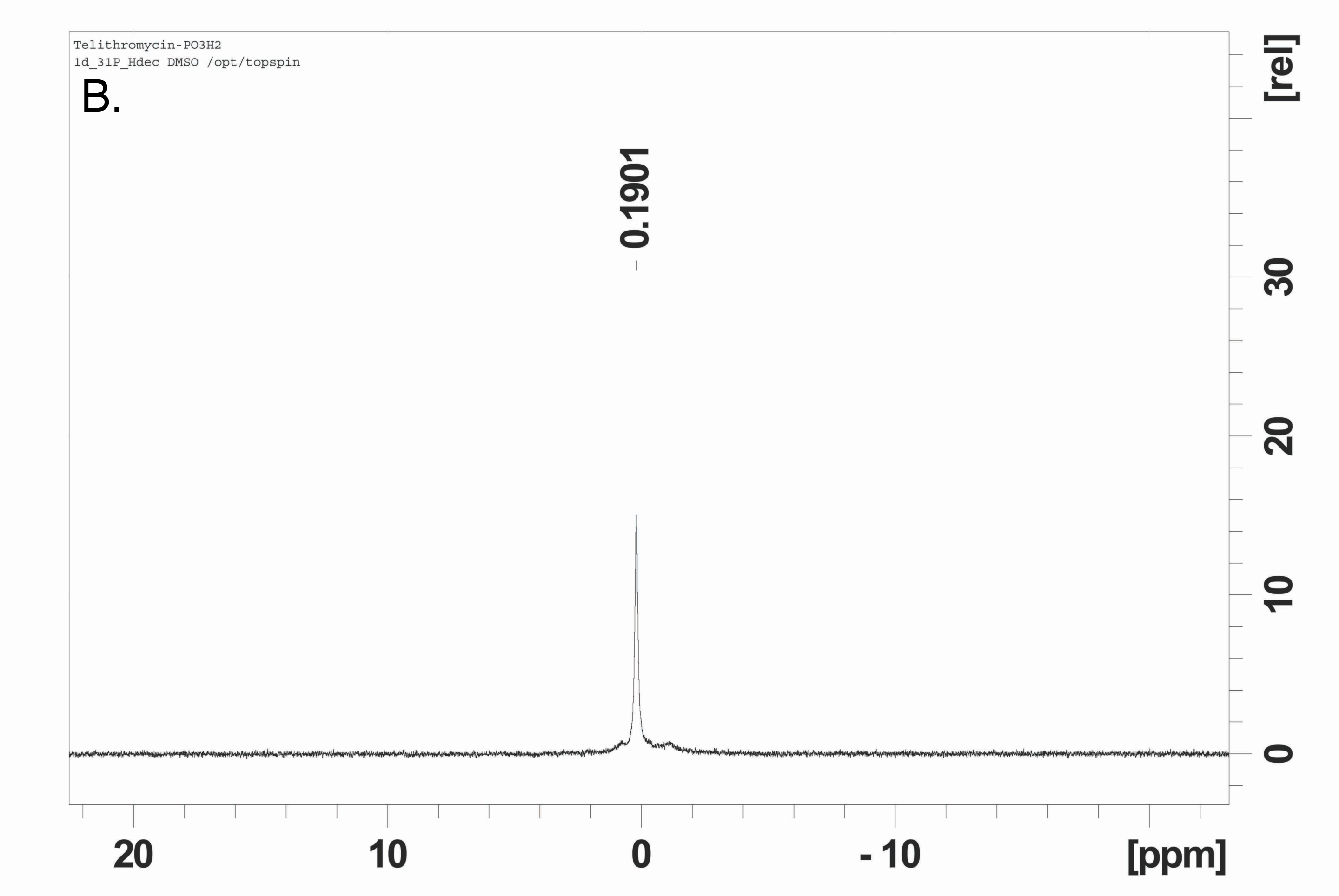

Supplement: Figure S4 — 31P – NMR spectra of telithromycin phosphate. (TIF) [file pone.0034953.s005.tif]

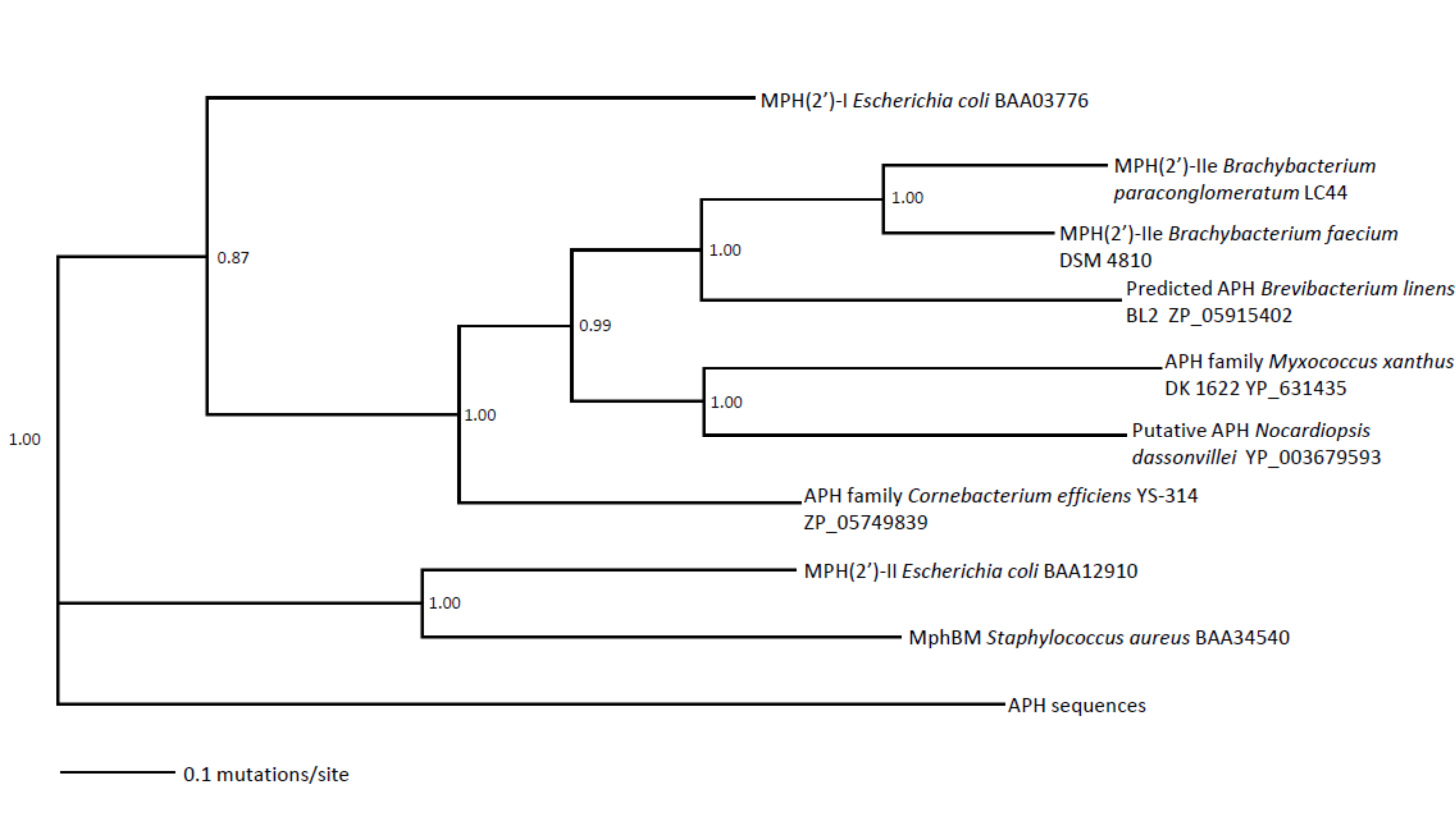

Supplement: Figure S5 — Phylogenetic Analysis of MPH/MPH-like proteins. Aminoglycoside Phosphotransferases (APHs) were collapsed into a single branch (APH sequences) which was used as an outgroup in this analysis of amino acid sequence. Note that the scale bar represents 0.1 mutations/site. (TIF) [file pone.0034953.s006.tif]
