# Supplementary material for: Antibiotic Resistance Is Prevalent in an Isolated Cave Microbiome
Source: PLoS One. 2012 Apr 11;7(4):e34953. doi: 10.1371/journal.pone.0034953 (PMC3324550; doi:10.1371/journal.pone.0034953)
Supplement: Table S1 — Media Used for Initial Bacterial Cultivation. (DOCX) [file pone.0034953.s007.docx]

**Table S1. Media Used for Initial Bacterial Cultivation^a^**

| **Designation**^b^ | **Carbon Source** | **Additives** | **Antibiotics** |
| --- | --- | --- | --- |
| DW | None added | NPS^c^ | +/- Ampicillin |
| F | Fulvic acids^d^ |  |  |
| H | Humic acids^d^ |  |  |
| F/C | Fulvic acids | CaCO_3_^e^ | =/- Tetracycline/Nalidixic acid or Ampicillin |
| H/C | Humic acids | CaCO_3_ |  |
| P/CAS | Sodium pyruvate (25 mM) | CaCO_3_ |  |
| VM/C/C1 | Calcium formate (20 mM), methanol (0.2%) | VM^f^, CaCO_3_ | +/- Ampicillin |
| VM/NPS/P/CAS | Sodium pyruvate (25 mM), CAS amino acids (0.03%) | VM, NPS^f^ | +/- Ampicillin or Chloramphenicol |
| VM/C/NPS/P/CAS | Sodium pyruvate (25 mM), CAS amino acids (0.03%) | VM, NPS, CaCO_3_ |  |

^a^ Given as Litre^-1^

^b^ All media used agarose as a gelling agent and was prepared with de-ionized water containing ~ 2 mg/L total organic carbon (TOC)

^c^ Trace nitrogen/phosphorous/sulfur (NPS) were added as follows: 64.0 mg Na_2_HPO_4_.7H_2_O, 15.0 mg KH_2_PO_4_, 5.0 mg NH_4_Cl and 2.4 mg MgSO_4_.

^d^ Fulvic and humic acids were extracted from commercially available potting soil as follows: 100 g of soil was mixed with 400 ml of water and the pH was adjusted to 7.0 using 1 M NaOH. This slurry was allowed to stir for 4 hours, after which the supernatant was collected by settling of the particulate matter followed by decanting of the supernatant. This supernatant was then successively filtered, first through cheesecloth and then through increasingly fine Whatman (grade 6) filter papers. The pH of the clarified supernatant was dropped to 1.0 using 1 M HCl and stirred gently for 4 hours, allowing the humic acids to precipitate. Centrifugation at 10,000 x *g* was then used to separate the supernatant (fulvic acids) from the the pellet (humic acids). The fulvic acid fraction was adjusted to pH 7.0 using 1 M NaOH, and the humic acid pellet was resuspended to the same volume as the fulvic acid fraction and also adjusted to pH 7.0 using NaOH. The final concentration of each was calculated using a Shimadzu TOC.

^e^ 4 g calcium carbonate (CaCO_3_) fine powder was added.

^f^ Added vitamins and minerals was based on Wolfe’s vitamin/mineral supplement.
