# Supplementary material for: Antibiotic Resistance Is Prevalent in an Isolated Cave Microbiome
Source: PLoS One. 2012 Apr 11;7(4):e34953. doi: 10.1371/journal.pone.0034953 (PMC3324550; doi:10.1371/journal.pone.0034953)
Supplement: Table S2 — List of strains used in this study. (DOCX) [file pone.0034953.s008.docx]

**Table S2. List of strains used in this study.**

| **Isolate** | **Sample site** | | **Isolation media** | **Antibiotics *** | | | **16S ID** | | **Gram** | |
| --- | --- | --- | --- | --- | --- | --- | --- | --- | --- | --- |
| LC 3 | LCECE | | FC |  | | | *Rhizobium sp.* B282 | | - | |
| LC 4 | LCECE | | FC |  | | | *Bosea sp.* PD 23 | | - | |
| LC 5 | LCECE | | FC |  | | | *Devosia neptuniae* | | - | |
| LC 9 | LCECE | | VM/C/NPS/P/CAS |  | | | *Streptomyces mauvecolor* | | + | |
| LC 10 | LCECE | | VM/C/NPS/P/CAS |  | | | *Devosia neptuniae* | | - | |
| LC 11 | LCECE | | VM/C/NPS/P/CAS |  | | | *Ensifer adhaerens* | | - | |
| LC 12 | LCECE | | VM/C/NPS/P/CAS |  | | | *Sphingobium yanoikuyae* | | - | |
| LC 13 | LCECE | | VM/C/NPS/P/CAS |  | | | *Ensifer adhaerens* | | - | |
| LC 14 | LCECE | | VM/C/NPS/P/CAS |  | | | *Ensifer adhaerens* | | - | |
| LC 17 | LCDS1B | | VM/C/NPS/P/CAS |  | | | *Massilia timonae* | | - | |
| LC 18 | LCDS1B | | VM/C/NPS/P/CAS |  | | | *Agrococcus jenensis* | | + | |
| LC 19 | LCDS1B | | VM/C/NPS/P/CAS |  | | | *Ochrobactrum intermedium* | | - | |
| LC 23 | LCDS1B | | C/P/CAS |  | | | *Betaproteobacterium* HI-B12 | | - | |
| LC 29 | LCECE | | VM/NPS/P/CAS |  | | | *Streptomyces flaveus* | | + | |
| LC 30 | LCECE | | HC |  | | | *Streptomyces anulatus* | | + | |
| LC 31 | LCECE | | H |  | | | *Streptomyces anulatus* | | + | |
| LC 34 | LCECE | | VM/NPS/P/CAS |  | | | *Agrobacterium tumefaciens* | | - | |
| LC 36 | LCECE | | VM/NPS/P/CAS |  | | | *Brevundimonas vesicularis* | | - | |
| LC 37 | LCECE | | VM/NPS/P/CAS |  | | | *Acinetobacter calcoaceticus* | | - | |
| LC 38 | LCECE | | VM/NPS/P/CAS |  | | | *Ochrobactrum anthropi* | | - | |
| LC 44 | LCDS1B | | VM/NPS/P/CAS |  | | | *Brachybacterium paraconglomeratum* | | + | |
| LC 54 | LCECE | | HC |  | | | *Ensifer adhaerens* | | - | |
| LC 65 | LCDS1B | | NPS |  | | | *Ochrobactrum anthropi* | | - | |
| LC 69 | LCECE | | NPS |  | | | *Arthrobacter sp*. M4 | | + | |
| LC 70 | LCDS1B | | H |  | | | *Microbacterium sp.* AC35 | | + | |
| LC 74 | LCDS1B | | H |  | | | *Rhodococcus erythropolis* | | + | |
| LC 75 | LCECE | | H |  | | | *Rhodococcus erythropolis* | | + | |
| LC 77 | LCECE | | H |  | | | *Sphingomonas sp.* HI-K4 | | - | |
| LC 78 | LCECE | | H |  | | | *Streptomyces mauvecolor* | | + | |
| LC 79 | LCECE | | H |  | | | *Nocardia asteroides* | | + | |
| LC 81 | LCECE | | H |  | | | *Sphingopykis sp*. DG892 | | - | |
| LC 83 | LCECE | | H |  | | | *Sphingomonas sp*. HI-K4 | | - | |
| LC 85 | LCECE | | F |  | | | *Bosea thiooxidans* | | - | |
| LC 86 | LCECE | | F |  | | | *Bosea thiooxidans* | | - | |
| LC 87 | LCECE | | F |  | | | *Bosea thiooxidans* | | - | |
| LC 92 | LCECE | | F |  | | | *Bosea thiooxidans* | | - | |
| LC 103 | LCECE | | VM/C/C1 |  | | | *Mesorhizobium plurifarium* | | - | |
| LC 104 | LCECE | | VM/C/C1 |  | | | *Alphaproteobacterium* clone RR4A6 | | - | |
| LC 112 | LCDS1B | | F |  | | | Uncultured *Roseomonas sp* | | - | |
| LC 113 | LCDS1B | | F |  | | | Uncultured *Roseomonas sp* | | - | |
| LC 143 | LCEA1 | | FC |  | | | *Arthrobacter oxydans* | | + | |
| LC 145 | LCEA1 | | FC |  | | | *Rhizobium galegae* | | - | |
| LC 148 | LCEA1 | | FC |  | | | *Rhizobium sp*. ORS 1465 | | - | |
| LC 153 | LCEA1 | | FC |  | | | *Kocuria rosea* | | + | |
| LC 163 | LCEA1 | | DW |  | | | *Ensifer adhaerens* | | - | |
| LC 230 | LCEA1 | | VM/C/NPS/P/CAS |  | | | *Paenibacillus lautus* | | + | |
| LC 231 | LCEA1 | | VM/C/NPS/P/CAS |  | | | *Paenibacillus lautus* | | + | |
| LC 236 | LCEA1 | | VM/C/NPS/P/CAS |  | | | *Pseudoxanthomonas mexicana* | | - | |
| LC 238 | LCEA1 | | VM/C/NPS/P/CAS |  | | | *Naxibacter varians* | | - | |
| LC 241 | LCEA1 | | PCAS |  | | | *Sphingomonas pseudosanguinis* | | - | |
| LC 242 | LCEA1 | | PCAS |  | | | *Paenibacillus lautus* | | + | |
| LC 249 | LCECE | | VM/C/NPS/P/CAS |  | | | *Microbacterium esteraromaticum* | | + | |
| LC 263 | LCEA1 | | PCAS |  | | | *Pseudomonas stutzeri* | | - | |
| LC 265 | LCEA1 | | PCAS |  | | | *Paenibacillus lautus* | | + | |
| LC 268 | LCDS1B | | P/C/CAS |  | | | *Massilia timonae* | | - | |
| LC 278 | LCEA1 | | VM/C/C1 | AMP | | | *Microbacterium phyllosphaerae* | | + | |
| LC 285 | LCEA1 | | FC | TN | | | *Brevibacterium casei* | | + | |
| LC 289 | LCEA1 | | FC | AMP | | | *Arthrobacter sp*. Ellin159 | | + | |
| LC 363 | LCECE | | DW |  | | | *Sphingopyxis alaskensis* | | - | |
| LC 364 | LCEA1 | | DW |  | | | *Ochrobactrum anthropi* | | - | |
| LC 365 | LCEA1 | | VM/NPS/P/CAS | AMP | | | *Actinobacterium* CH21i | | + | |
| LC 368 | LCEA1 | | VM/NPS/P/CAS | AMP | | | *Microbacterium hominis* | | + | |
| LC 371 | LCEA1 | | VM/NPS/P/CAS |  | | | *Pseudomonas stutzeri* | | - | |
| LC 378 | LCEA1 | | VM/NPS/P/CAS |  | | | *Mesorhizobium mediterraneum* | | - | |
| LC 379 | LCEA1 | | DW |  | | | *Sphingomonas sp.* HI-K4 | | - | |
| LC 383 | LCDS1B | | HC |  | | | *Sphingomonas dokdonensis* | | - | |
| LC 384 | LCDS1B | | HC |  | | | *Ensifer adhaerens* | | - | |
| LC 387 | LCEA1 | | VM/NPS/P/CAS | AMP | | | *Tetrathiobacter kashmirensis* | | - | |
| LC 390 | LCEA1 | | VM/NPS/P/CAS | AMP | | | *Leucobacter alluvii* | | + | |
| LC 391 | LCEA1 | | DW |  | | | *Variovorax sp*. HI-I4 | | - | |
| LC 392 | LCEA1 | | VM/C/NPS/P/CAS |  | | | *Bosea sp*. CRIB-12 | | - | |
| LC 400 | LCEA1 | | VM/C/NPS/P/CAS | AMP | | | *Pseudomonas stutzeri* | | - | |
| LC 401 | LCEA1 | | VM/C/NPS/P/CAS | AMP | | | *Dietzia maris* | | + | |
| LC 404 | LCEA1 | | VM/C/NPS/P/CAS | AMP | | | *Brevundimonas aurantiaca* | | - | |
| LC 409 | LCEA1 | | VM/C/NPS/P/CAS | AMP | | | *Microbacterium esteraromaticum* | | + | |
| LC 411 | LCDS1B | | HC |  | | | *Sphingomonas sp*. HI-K4 | | - | |
| LC 412 | LCECE | | VM/C/NPS/P/CAS |  | | | *Rhodococcus erythropolis* | | + | |
| LC 415 | LCEA1 | | VM/NPS/P/CAS | AMP | | | *Rhodococcus erythropolis* | | + | |
| LC 421 | LCEA1 | | FC |  | | | *Achromobacter sp*. LMG 5911 | | - | |
| LC 424 | LCEA1 | | VM/NPS/P/CAS | AMP | | | *Microbacterium sp.* AC35 | | + | |
| LC 425 | LCEA1 | | VM/NPS/P/CAS |  | | | *Pseudoxanthomonas mexicana* | | - | |
| LC 458 | LCEA1 | | H |  | | | *Achromobacter spanius* | | - | |
| LC 469 | LCEA1 | | F |  | | | *Microbacterium esteraromaticum* | | + | |
| LC 485 | LCEA1 | | PCAS |  | | | *Brevibacterium casei* | | + | |
| LC 486 | LCEA1 | | PCAS |  | | | *Brevibacterium casei* | | + | |
| LC 498 | LCECE | | VM/NPS/P/CAS |  | | | *Ochrobactrum anthropi* | | - | |
| LC 499 | LCECE | | VM/NPS/P/CAS |  | | | *Ensifer adhaerens* | | - | |
| LC 500 | LCECE | | VM/NPS/P/CAS |  | | | *Sphingomonas yanoikuyae* | | - | |
| LC 506 | LCDS1B | | P/C/CAS |  | | | *Ochrobactrum intermedium* | | - | |
| LC 507 | LCDS1B | | P/C/CAS |  | | | *Microbacterium esteraromaticum* | | + | |
| LC 508 | LCECE | | VM/C/NPS/P/CAS |  | | | *Devosia neptuniae* | | - | |
| LC 509 | LCEA1 | | FC |  | | | *Micrococcus luteus* CV39 | | + | |
| LC 510 | LCECE | | VM/NPS/P/CAS |  | | | *Acinetobacter calcoaceticus* | | - | |
| LC 511 | LCDS1B | | VM/C/NPS/P/CAS |  | | | *Brevundimonas vesicularis* | | - | |
| LC 513 | LCDS1B | | VM/C/NPS/P/CAS |  | | | *Methylobacterium lusitanum* | | - | |
| VM = Vitamins Minerals | | | F = Fulvic acid |  | | ** Antibiotic added to the media* | |  | |  |
| P = Pyruvate | | | H = Humic acid |  | | *AMP = Ampicillin* | |  | |  |
| C = CaCO3 | |  | DW = Distilled water |  | | *TN = Trimethoprim* | |  | |  |
| C1 = Methanol/formate | | | NPS = Nitrogen/Phosphate/Sulfur | | |  | |  | |  |
|  | |  | CAS= CAS Amino acids | |  |  | |  | |  |
