# Supplementary material for: Antibiotic Resistance Is Prevalent in an Isolated Cave Microbiome
Source: PLoS One. 2012 Apr 11;7(4):e34953. doi: 10.1371/journal.pone.0034953 (PMC3324550; doi:10.1371/journal.pone.0034953)
Supplement: Table S3 — Telithromycin Inactivation in Cave Isolates. (DOCX) [file pone.0034953.s009.docx]

**Table S3. Telithromycin Inactivation in Cave Isolates.** The strains were grown in presence of 20 µg/ml drug for 5 days at 30 °C. Analysis of the clarified culture supernatant by LC-MS* indicated a shift of 162 Da (for *Streptomyces* isolates) and 80 Da (for LC44) in comparison to the telithromycin reference, suggestive of mono-glycosylation and phosphorylation respectively.

| Strain | Telithromycin *m/z*  [M+H]^+^ | Retention Time (min) | Telithromycin Inactivation Product *m/z* [M+H]^+^ | Retention Time (min) | Difference  *m/z* |
| --- | --- | --- | --- | --- | --- |
| *Streptomyces flaveus* (LC29) | 813.0 | 13.2 | 974.6 | 12.9 | 161.6 |
| *Streptomyces anulatus* (LC30) | 813.0 | 13.2 | 974.6 | 12.9 | 161.6 |
| *Streptomyces anulatus* (LC31) | 813.0 | 13.2 | 974.6 | 12.9 | 161.6 |
| *Brachybacterium paraconglomeratum* (LC44) | 812.6 | 13.1 | 892.6 | 13.3 | 80.0 |

* The reverse phase HPLC conditions are as follow: isocratic 5% solvent B (0.05% formic acid in acetonitrile), 95% solvent A (0.05% formic acid in water) over 5 min, followed by a linear gradient to 97% B over 20 min at a flow rate of 1 ml/min.
