# Supplementary material for: Antibiotic Resistance Is Prevalent in an Isolated Cave Microbiome
Source: PLoS One. 2012 Apr 11;7(4):e34953. doi: 10.1371/journal.pone.0034953 (PMC3324550; doi:10.1371/journal.pone.0034953)
Supplement: Table S4 — Tandem mass spectrometry analysis of ring-opened daptomycin by Paenibacillus lautus LC231. (DOCX) [file pone.0034953.s010.docx]

**Table S4. Tandem mass spectrometry analysis of ring-opened daptomycin by *Paenibacillus lautus* LC231.** The observed ions are highlighted in bold.


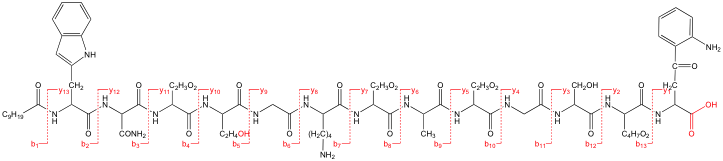


| **Ions** | **m/z** | **Ions** | **m/z** |
| --- | --- | --- | --- |
| **b13** | 1430.64 | y13 | 1484.59 |
| **b12** | 1287.59 | **y12** | 1298.51 |
| **b11** | 1200.44 | **y11** | 1184.47 |
| **b10** | 1143.53 | **y10** | 1069.44 |
| **b9** | 1028.30 | **y9** | 968.40 |
| **b8** | 957.17 | **y8** | 911.37 |
| **b7** | 842.44 | **y7** | 797.29 |
| **b6** | 728.24 | **y6** | 682.27 |
| **b5** | 671.34 | **y5** | 611.23 |
| **b4** | 570.29 | **y4** | 496.25 |
| **b3** | 455.27 | **y3** | 439.18 |
| **b2** | 341.22 | **y2** | 352.15 |
| b1 | 155.14 | y1 | 209.09 |
