# Supplementary material for: Antibiotic Resistance Is Prevalent in an Isolated Cave Microbiome
Source: PLoS One. 2012 Apr 11;7(4):e34953. doi: 10.1371/journal.pone.0034953 (PMC3324550; doi:10.1371/journal.pone.0034953)
Supplement: Table S5 — Chloramphenicol Inactivation in Cave Strains. (DOCX) [file pone.0034953.s011.docx]

**Table S5. Chloramphenicol Inactivation in Cave Strains.** The strains were grown in presence of 20 µg/ml drug for 5 days at 30°C. Clarified cultured supernatant was used for LC-MS analysis. Based on shift in retention time and *m/z* ratio, modification was inferred to be mono-acetylation of chloramphenicol.

| Strain | Chloramphenicol *m/z*  [M-H]^-^ | Retention Time (min) | Chloramphenicol Inactivation Product *m/z*  [M-H]^-^ | Retention Time (min) | Difference  *m/z* |
| --- | --- | --- | --- | --- | --- |
| *Ochromobactrum intermedium* (LC19) | 321.3 | 5.6 | 363.4 | 6.3 | 42.1 |
| *Agrobacterium tumefaciens* (LC34) | 321.3 | 5.6 | 363.3 | 6.3 | 42.0 |
| *Ochromobactrum intermedium* (LC506) | 321.3 | 5.6 | 363.1 | 6.3 | 41.8 |
