# Supplementary material for: Antibiotic Resistance Is Prevalent in an Isolated Cave Microbiome
Source: PLoS One. 2012 Apr 11;7(4):e34953. doi: 10.1371/journal.pone.0034953 (PMC3324550; doi:10.1371/journal.pone.0034953)
Supplement: Table S6 — 1H Chemical Shifts of Telithromycin and Inactivated Product of B. paraconglomeratum LC44 in DMSO-d6 (ppm). (DOCX) [file pone.0034953.s012.docx]

**Table S6. ^1^H Chemical Shifts of Telithromycin and Inactivated Product of *B. paraconglomeratum* LC44 in DMSO-d_6_ (ppm).**

| Proton | Telithromycin | Telithromycin product |
| --- | --- | --- |
| 2 | 4.03 | 4.03 |
| 2-CH_3_ | 1.21 | 1.22 |
| 4 | 3.09 | 3.05 |
| 4-CH_3_ | 1.21 | 1.21 |
| 5 | 4.03 | 4.03 |
| 6-CH_3_ | 1.19 | 1.18 |
| 6-OCH_3_ | 2.44 | 2.43 |
| 7a | 1.66 | 1.69 |
| 7b | 1.62 | 1.65 |
| 8 | 2.45 | 2.42 |
| 8-CH_3_ | 1.13 | 1.11 |
| 10 | 3.15 | 3.16 |
| 10-CH_3_ | 0.88 | 0.88 |
| 11 | 3.44 | 3.44 |
| 12-CH_3_ | 1.49 | 1.48 |
| 13 | 4.76 | 4.75 |
| 14a | 1.73 | 1.73 |
| 14b | 1.56 | 1.56 |
| 15 | 0.76 | 0.76 |
| 17a | 3.56 | 3.55 |
| 17b | 3.47 | 3.47 |
| 18a,b | 1.48 | 1.48 |
| 19a,b | 1.74 | 1.74 |
| 20a | 4.00 | 3.99 |
| 20b | 3.99 | 3.99 |
| 21 | 7.71 | 7.70 |
| 23 | 7.77 | 7.77 |
| 25 | 8.93 | 8.92 |
| 26 | 8.38 | 8.38 |
| 27 | 7.35 | 7.35 |
| 28 | 8.05 | 8.05 |
| 1′ | 4.17 | 4.33 |
| 2′ | 3.06 | 3.71 |
| 3′ | 2.48 | 3.62 |
| 3′-N(CH_3_)_2_ | 2.21 | 2.42 |
| 4′a | 1.63 | 1.26 |
| 4′b | 1.11 | 1.17 |
| 5′ | 3.51 | 3.08 |
| 5′-CH_3_ | 1.14 | 1.19 |
