# Supplementary material for: Antibiotic Resistance Is Prevalent in an Isolated Cave Microbiome
Source: PLoS One. 2012 Apr 11;7(4):e34953. doi: 10.1371/journal.pone.0034953 (PMC3324550; doi:10.1371/journal.pone.0034953)
Supplement: Table S7 — 13C Chemical Shifts of Telithromycin and Inactivated Product of B. paraconglomeratum LC44 in DMSO-d6 (ppm). (DOCX) [file pone.0034953.s013.docx]

**Table S7. ^13^C Chemical Shifts of Telithromycin and Inactivated Product of *B. paraconglomeratum* LC44 in DMSO-d_6_ (ppm).**

| Carbon | Telithromycin | Telithromycin product |
| --- | --- | --- |
| 1 | 169.62 | 169.8 |
| 2 | 50.22 | 50.37 |
| 2-CH_3_ | 15.73 | 15.95 |
| 3 | 203.91 | 203.7 |
| 4 | 46.88 | 52.27 |
| 4-CH_3_ | 13.97 | 14.13 |
| 5 | 78.5 | 79.0 |
| 6 | 77.73 | 77.87 |
| 6-CH_3_ | 19.44 | 19.59 |
| 6-OCH_3_ | 49.21 | 49.33 |
| 7 | 38.69 | 38.48 |
| 8 | 44.47 | 44.56 |
| 8-CH_3_ | 17.75 | 17.77 |
| 9 | 216.01 |  |
| 10 | 38.34 | 38.40 |
| 10-CH_3_ | 13.47 | 13.44 |
| 11 | 59.8 | 59.90 |
| 12 | 81.98 | 82.1 |
| 12-CH_3_ | 14.3 | 14.12 |
| 13 | 76.61 | 76.50 |
| 14 | 21.87 | 21.85 |
| 15 | 10.33 | 10.40 |
| 16 | 156.25 | 156.24 |
| 17 | 42.12 | 42.13 |
| 18 | 23.71 | 23.84 |
| 19 | 27.88 | 27.89 |
| 20 | 45.81 | 45.95 |
| 21 | 138.19 | 138.19 |
| 22 | 137.61 | 137.60 |
| 23 | 116.48 | 116.60 |
| 24 | 130.33 | 129.70 |
| 25 | 145.64 | 145.91 |
| 26 | 147.06 | 147.30 |
| 27 | 123.57 | 123.57 |
| 28 | 131.08 | 131.10 |
| 1′ | 103.75 | 101.80 |
| 2′ | 70.19 | 71.0 |
| 3′ | 64.48 | 67.36 |
| 3′-N(CH_3_)_2_ | 40.3 | 39.70 |
| 4′ | 29.96 | 28.87 |
| 5′ | 68.39 | 67.95 |
| 5’-CH_3_ | 21.06 | 20.80 |

The presence of a phosphate group was confirmed by the observation of a single resonance at 0.19 ppm in ^31^P NMR spectra and as well by downfield chemical shifts for the protons at position 1’, 2’ and 3’- neighboring the phosphate group.
